# Improving short-term water demand forecasting using evolutionary algorithms

**DOI:** 10.1038/s41598-022-17177-0

**Published:** 2022-08-08

**Authors:** Justyna Stańczyk, Joanna Kajewska-Szkudlarek, Piotr Lipiński, Paweł Rychlikowski

**Affiliations:** 1 Institute of Environmental Engineering, Wroclaw University of Environmental and Life Sciences, 24 Grunwaldzki Square, 50-363 Wrocław, Poland; 2 Institute of Computer Science, University of Wroclaw, 15 Joliot-Curie Street, 50-383 Wrocław, Poland

**Keywords:** Environmental sciences, Engineering, Mathematics and computing

## Abstract

Modern solutions in water distribution systems are based on monitoring the quality and quantity of drinking water. Identifying the volume of water consumption is the main element of the tools embedded in water demand forecasting (WDF) systems. The crucial element in forecasting is the influence of random factors on the identification of water consumption, which includes, among others, weather conditions and anthropogenic aspects. The paper proposes an approach to forecasting water demand based on a linear regression model combined with evolutionary strategies to extract weekly seasonality and presents its results. A comparison is made between the author's model and solutions such as Support Vector Regression (SVR), Multilayer Perceptron (MLP), and Random Forest (RF). The implemented daily forecasting procedure allowed to minimize the MAPE error to even less than 2% for water consumption at the water supply zone level, that is the District Metered Area (DMA). The conducted research may be implemented as a component of WDF systems in water companies, especially at the stage of data preprocessing with the main goal of improving short-term water demand forecasting.

## Introduction

Water consumption prediction is one of the main goals in managing water supply infrastructure and water resources. Increasing urbanization, industrialization, population growth and unavoidable climate change cause the demand for drinking water to increase constantly^1^. Freshwater resources for water supply are constantly decreasing, and although we are demonstrating more and more conscious and pro-ecological attitudes in terms of water saving, studies show significant water scarcity of water globally and locally, especially in countries with dry climate^2^. Forecasting water demand over different time horizons is crucial in the management of water resources. Depending on the purpose of the observations, developed Water Demand Forecasting (WDF) systems allow predicting water consumption in short- and long-term perspectives. The aim of long-term analyses, considering the horizon of 20–30 years, is to support making decisions related to designing and developing water supply systems. Short-term simulations, usually hourly, daily, or weekly^3,4^, are used to optimize the work and energy costs of pump stations (Pump Scheduling Optimization, PSO)^5^ and to solve current operational problems^6^. Tiwari and Adamowski^7^ as well as Candelieri et al.^8^ also distinguish medium-term predicting with respect to weekly time ranges. It is used in supply network maintenance and developing failure prevention procedures. Future water consumption is always estimated based on historical time series. The methodology of their analyses consists in identifying the current pattern of water demand and predicting future consumption.

Apart from the purpose of water demand prediction that was mentioned above, its key aim is to ensure the appropriate amount of water to users in a long-term perspective, which is a difficult task, mainly in dry climate countries. This problem is especially intensified in large urban centers with a continuously increasing number of residents^9–11^. The analysis of the aquifer content that changes in time depending on external factors allows to determine their quantitative potential in terms of future water intake, water treatment and pumping it into the water supply system^12^. To be able to assess the sufficiency of water resources, it is necessary to implement procedures that are used in long-term forecasting.

Both short-term and long-term predictions require knowledge of the influence of factors that result in irregularities in water consumption from the water supply network. Short-term predictions require mainly the meteorological parameters analysis, whereas long-term forecasts require additionally analyzing demographic and economic factors^10^. The current weather conditions, geographical location, and socio-demographic aspects, referred to as anthropogenic^11^, determine the existence of hourly, daily, weekly or seasonal variability. The influence of anthropogenic factors is globally enhanced on holidays (Sundays, public holidays, and school holidays), in periods preceding religious holidays and locally, for example, during major cultural and leisure events.

One component of smart cities, an idea that is constantly being implemented and improved as part of the city vision of the future^13^ is Intelligent Water Systems (IWSs). Intelligence in the water sector, according to the Water Environment Federation, is evidenced by the use of advanced technologies in decision making and management. In practice, this means, among other things, the need to reduce operational costs, manage and mitigate risks, which should be served by risk assessment solutions, failure prediction, performance prediction and decision support systems^14^. Smart water supply systems take into consideration the variability in water consumption in the prediction sector, for systems of residential water saving^15^ and in solutions aimed at detecting abnormal states. Modelling their behavior requires advanced methods because of water supply system complexity^16^. The implementation of meteorological data enables to reduce the predicting error even by 11%^17^. The knowledge of the meteorological factors is necessary to obtain the best quality of water consumption prediction model^18^. Not taking into account the variability in water consumption in failure detecting systems results in an increased number of generated false alarms^19^. Including meteorological factors in the input data used for water consumption forecasts is therefore crucial, especially during short- and medium-term analyses^13,20,21^.

The purpose of the study was to create a methodology for extracting weekly seasonality in water consumption time series using evolutionary algorithms, which were implemented to improve the daily forecast accuracy. To the best of the authors' knowledge, this study is one of the few attempts to application of the evolutionary approach for water consumption time series implemented to improve prediction accuracy. The paper presents the results of short-term predictions of water consumption in one of the District Metered Area (DMA) zones located in Wroclaw (Poland), characterized by multifamily buildings. The applied models are based on evolutionary algorithms and on standard approach: Support Vector Regression (SVR), Multilayer Perceptron (MLP) and Random Forest (RF). Advanced methods in the field of computational intelligence are used in the research. These methods include evolutionary algorithms that discover seasonality, which is hard to determine when using simple methods due to the additional overlapping of, e.g., the development trends of DMA zones and annual seasonality. The implemented procedure allowed the authors to minimize the MAPE forecast error to even less than 2%, taking into account various case study variants. Regular evolutionary strategies may provide a tool that will support water demand prediction and thus improve the ongoing decision-making processes on the level of water supply networks managed and operating. The presented approach towards forecasting water demand levels, based on applying classical evolutionary strategies, shows that getting to know the factors determining water consumption variability is a key aspect. According to the research^22^, not only the analyses of meteorological parameters should be implemented in WDF models, but they should also take into consideration the periodicity of water consumption resulting from anthropogenic behavior, for instance, the different property characteristics, various household composition in terms of number and age, socioeconomic level and education level of the consumers. Finally, it may also become a component of systems that warn about abnormal states, such as failures, which are a widely analyzed aspect of managing water supply networks^23^.

The first section of the article contains a literature review concerning the methods used to predict water consumption. The following sections present the problem statement of the research, the methods as well as the results of the research conducted by the authors, and their interpretation and discussion. The final section of the paper provides some conclusions.

## Literature review of water demand forecasting methods

The optimization of water supply system operation is an important and up-to-date problem in civil engineering concerning increasingly scientific research^24,25^. The great progress in predicting water consumption that has been noted in recent years was caused by the intensive development and implementation of algorithms embedded in advanced computational intelligence. The subject of analyses and interpretation are not only historical hydraulic parameters, but also meteorological conditions.

The methods are currently applied for water demand forecasting have been presented in the form of a table (Table 1). Different characteristics for every case study and using various kinds of errors for evaluation cause that it is difficult to compare the model implemented by the authors. For this reason, the research compares the proprietary model with standard methods. Moreover, a review of the literature shows that appropriate techniques are rarely used in data preprocessing. Discrete wavelet transform (DWT) is most often used for this purpose^26^.

**Table 1:** Review of the water demand forecasting methodology.

Authors	Year	Prediction methods	Forecast horizon	Length of the time series
Alvisi and Franchini^27^	2014	MCP ANN Patt_for Hybrid^a^	Hourly demand over a time horizon of 24 h	3 years
Baker et al.^17^	2014	AHM Transfer/-noise^a^ MLR	Daily	6 years
Candelieri et al.^9,28^	2014 2015	SVM^a^ ANN	Daily	2 years
Chen and Boccelli^29^	2014	SARIMA	Daily	39 days
Kofinas et al.^30^	2014	WAM ANN^a^ ARIMA^a^	Monthly, seasonal	3 years
Romano and Kapelan^31^	2014	EANN	Daily, hourly	181 days
Tiwari et al.^32^	2015 2016	WBANN ELMW ANNB	Monthly Weekly Daily	3 years
Arandia et al.^33^	2016	SARIMA	Sub-hourly, hourly and daily water demand	10/19 months
Brentan et al.^6^	2017	SVM-AFS	Monthly Weekly Daily, hourly	1,5 year
Ghiassi et al.^10^	2017	DAN2^a^ FTDNN KNN	Monthly Weekly Daily	8 years
Duerr et al.^34^	2018	Regression models AR(1) ARIMA GAM Spatio-temporal (ST) gaussian process models GBM RF BART	Monthly	12 years
Kozłowski et al.^35^	2018	Phase Trend Method PTM Harmonic analysis	Hourly demand over a time horizon of 1 month	2 months
Xenochristou et al.^36^	2018	Random Forests	Daily	3 years
Ambrosio et al.^20^	2019	Committee machines^a^: MLP SVM ELM RF ANFIS GMDH	Hourly	33 months
Xu et al.^37^	2019	CDBESN^a^ SVR ESN CDBNN	Hourly	11 months
Guo et al.^38^	2020	SMWOA^a^ ASLWOA WMWOA WOA	Yearly demand for water resources over a time horizon of 5 years	13 years
Karamaziotis et al.^39^	2020	ARIMA^a^ ETS Theta Opt.Theta MAPA MLP Ensemble	Monthly	7 years
Smolak et al.^40^	2020	ET SVR RF^a^ ARIMA/ARIMAX Blind	Weekly Daily	51 days
Bata et al.^41,42^	2020	RT with SOM	Hourly Daily Weekly	4 months
Shirkoohi et al.^4^	2021	ANN with genetic algorithm	15-min	5 years and 23 months

^a^The best matching effects when compared to the others.

The knowledge of the nature of water consumption should also constitute the basis for expert systems warning about failures, so-called Leak Detection Systems (LDS), and identifying the location of leaks. Unfortunately, due to the complexity of these solutions, these issues are often neglected. According to Brentan et al.^6^, short-term prediction is the key aspect of the detection of abnormal states. These states may be identified at the moment when the estimated and observed measurement values of hydraulic parameters differ. As far as this category of applications of WDF is concerned, the ARIMA models and ANN were implemented in the past. Okeya et al.^43^ supplemented their research on leak detection with the WDF method, thus reducing the number of false alarms warning about potential failures. Wu et al.^44^ developed a solution based on the Cluster Analysis of historical flow rate measurements. According to the authors, clusterization-based methods may be effectively applied in the analysis of nonstationary time series. Apart from that, they also minimize the number of positive false alarms and they may be implemented to detect large flow leaks. To ensure that the application of water consumption prediction systems will improve the results of detecting water supply network failures, short- and medium-term analyses should be conducted based on data recorded at time steps not exceeding 15 min^8^.

## Research problem definition

As the literature review in the article indicates, none of the methods that have been used so far is universal enough to be used for water consumption forecasts under all water network operating conditions. The non-stationarity of time series, related to the variable character of water consumption and other factors that affect it, makes it necessary to determine the long-term trend, annual and weekly seasonality. The aforementioned conditions result in the necessity to implement analytical tools connected with the variability in water consumption as part of advanced WDF systems^45,46^.

Despite the fact that there are many scientific approaches to the problem of water consumption forecasting, none of them is universal enough to be directly implemented in various operating conditions of water supply networks. It is because of the spatial variability of the weather, the specific operational characteristics of water supply systems and their users. The most frequently mentioned problems related to a reliable comparison of the quality of forecasting models include: different characteristics for every case study, forecasting horizon, size and types of samples, demand periodicity, and various, disproportionate forecast errors^5,18^. In addition, creating long-term forecasts is subject to bigger errors than short-term forecasts, i.e., daily forecasts are more reliable than weekly ones^40^.

The key issues raised in 6-year research concern the creation of a reliable forecasting model for water consumption based on measurements carried out on the actual water supply network. The obtained time series are difficult to interpret due to the fact that according to the classification, it is a large water supply company with many inhabitants and additionally students in the October-June/July period, which generates dynamic seasonality. According to the data of the Statistical Office in Wroclaw, approximately 120 thousand students resided in Wroclaw, which accounts for nearly 19% of the whole population^47^. During holidays, most of these students leave the city and return in October to continue education. Additionally, in July and August also permanent residents of Wroclaw are out of town on holiday. This phenomenon was highlighted in the works by Candelieri and Archetti^28^ and Candelieri et al.^9^ and it justifies considering different characteristics for holiday periods in predicting the consumption of tap water. The analyzed area is specific mainly because while other studies indicate an increased water consumption during the summer period^48^, in Wroclaw, a significant decrease is observed Table 1, presented in the previous section, shows the existence of many analytical approaches used in water demand forecasting. Therefore, an evolutionary approach was proposed to identify seasonality in the time series and the created model was compared with the standard methods (SVR, MLP, RF, and CART).

The input information for short-term forecasting, apart from water consumption, usually describes the weather conditions. Opinions on the impact of individual meteorological parameters on the quality of the model are strongly divided. Some scientists do not take them into account at all^5,37,45,49^, although the literature review by Sebri^18^ indicates that models of better accuracy were obtained for short-term and medium-term forecasts using meteorological data. Typically, the input base is based on weather data including air temperature, occurrence of precipitation, and air humidity. As far as air humidity is concerned, literature reports are contradictory, because some studies show the greatest dependence of water consumption on air humidity^50^, while Ambrosio et al.^20^ proved that including this parameter in the input data significantly worsens the prediction quality. The quality changes of the model are analyzed in this research, taking into account various input data.

Comprehensive knowledge of the problem results in improving the prediction systems in the analyzed DMA zones, however, it should be noted that both water consumption and meteorological time series are burdened with noise and errors, so working with their use is not a simple process^5^. Models based on actual measurement data are subject to uncertainty resulting from deficiencies caused, among others, by failure of measuring devices, meanwhile, reliable long-term forecasts are based on long-term measurement sequences^38^. According to the research^42^, for short-term forecasting, a 4-month measurement series is sufficient to obtain reliable water demand forecasts. Another goal of the research is the analysis of critical events in the operation of the water supply network, which largely contributed to the deterioration of the quality of prediction and the development of weekly periodicity patterns in water demand.

## Materials and methods

### Study area and datasets preparation

The input data for the model, related both to the hydraulic parameters of water flow inside water supply networks and weather elements, were obtained from the monitoring service of the Municipal Water and Sewage Company S.A. in Wroclaw, MPWiK (Lower Silesian Region, Poland). The water supply network of Wroclaw consists of more than 30 DMAs equipped with more than 80 measurement points. The analyzed period covered the daily time series from September 2014 to May 2020.

Water flow supplied to the area was measured with the use of an electromagnetic flow meter that records data at 10 min time intervals. It is installed in the zone measurement well in the multifamily building DMA. Time series recorded with a time step of 10 min are part of a reporting and network control system. A GSM module is used to transmit data to the water supply network operator in real time. Water is supplied to the discussed DMA through a water supply pipe of the diameter of DN500. The analyzed area is inhabited by more than 22 thousand people. To eliminate additional factors that might influence the prediction, data concerning abnormal situations, resulting, among others, from pipe failures and temporary breaks in data recording, were removed from the time series. During such events, zero pressure and flow are recorded.

The automatic meteorological station that provided the measurements is located in the city center. Similarly as in other large urban and industrial agglomerations, land development and land use lead to an Urban Heat Island forming, which results in the modification of temperature and precipitation in the city. The measured basic meteorological parameters included: air humidity and temperature, atmospheric pressure, wind speed, solar irradiation, and precipitation. The data is sent to a datalogger via a wireless GSM/GPRS connection. Although the automatic measurement station was programmed for sampling at 1-min intervals, the analyses used average values and daily sums of the particular meteorological parameters. This procedure was used because weather conditions measured with such a high-frequency measurement interval are not a factor in the ongoing variability of water use. The variation in minutes of weather is never significant in short-term prediction of water demand^51^.

To maintain the consistency of the analyses and the subsequent interpretation of the results, both during the creation of the model using standard methods and the evolutionary approach, the same datasets were used. To avoid the overfitting phenomenon, 10 random datasets were selected from the databases containing information on water demand and meteorological parameters. Each time, the training dataset was 365 days of measurement, while the testing set was 30 successive days. In some experiments, in addition to the data containing water consumption and meteorological parameters, characteristic variables were added to the input dataset. Most often, additional variables contain information about the day of the week, split into working days and weekends^9^. The authors' research included one water consumption of the last two weeks (history0—previous 7 days and history1—7 days before history0) and one hot encoding of the day of the week.

### Reference methods

In this article, several standard machine learning algorithms were used. To make this article self-contained, a very brief review of the implemented methods was presented. More details about these methods in general can be found in Murphy^52^ and Goodfellow et al.^53^.

#### Support vector regression

The idea of Support Vector Regression is similar to Support Vector Machines, but SVR concerns regression problems, while SVM—classification problems. SVR is a type of regressor, but more tolerant to outliers than regular linear regression as it does not count as error values which are close enough to the correct value. It can model nonlinear dependencies by mapping the original data space to a new data space, usually of a higher dimension, by some nonlinear kernel functions and using the kernel trick, which simplifies the transformation by allowing to express the inner products in the new data space by some precomputed values related to the kernels. In our experiments, various kernels were used, such as the linear kernel, the polynomial kernel, the sigmoid kernel or the RBF kernel, each of them with a different parameter setting leading to the best performance on a selected dataset, and the best results were obtained with the simplest variant (the linear kernel).

#### A multilayer perceptron

Nowadays, artificial neural networks are one of the most promising and most popular methods in machine learning. They are loosely inspired by biological neural networks, but can be treated as a way of describing a computation of a function values using well-defined, differentiable operations: matrix multiplication and addition, function composition, and some nonlinear functions (such as tanh, or Rectified Linear Units, ReLU). The authors used the popular architecture of a multilayer perceptron with two layers of hidden neurons (as more hidden layers did not lead to significant improvements in the computational experiments, only to overfitting). Several hidden layer sizes were tested in this research.

#### Linear regression

In this approach, the authors assume that the predicted value is a linear combination of all features (all categorical features, such as days of the week, should be represented by several binary variables, using one-hot encoding). The optimal values of the coefficients can be found using linear algebra methods or gradient-based methods (such as Stochastic Gradient Descent, SGD). LR is as one of the simplest machine learning methods.

#### Classification and regression trees (CART)

The authors construct a tree which describes in a concise manner what conditions the feature values should meet to give certain output values. CART method allows to describe very precisely the training set, but if it is necessary to achieve good generalization properties, some pruning techniques should be used.

#### Random Forests

Random Forests are an example of ensemble learning. In these methods, the authors try to combine the results of many rather weak classifiers/regressors. In random forests, CART trees (with limited depth) were used. These trees use different subsets of learning examples and different subsets of features as well. The authors used two depth limits: 5 and 10.

For all these methods, the scikit-learn library for Python^54^ programming language was used.

### Evaluating forecast accuracy

The main aim is to minimize the objective function, which is the Mean Absolute Percentage Error (MAPE) (using to evaluate models’ accuracy, among others by Bakker et al.^17^; Candelieri and Archetti^28^; Candelieri et al.^9^; Xu et al.^37^) of the forecasts obtained with use of machine learning. This kind of error is scale-independent, so it is especially recommended for use in the predictive model evaluation^18^. MAPE may be described by the following equation^55^:

1 $$MAPE=\frac{1}{n}\sum_{t=1}^{n}\frac{\left|{\widehat{y}}_{t}-{y}_{t}\right|}{{y}_{t}}\cdot 100 \%$$

where: $n$—the size of the sample, ${\widehat{y}}_{t}$—the value predicted by the model for time point $t$, ${y}_{t}$—the value observed at time point $t$.

## Evolutionary approach

Most of the prediction models require stationary time series to provide accurate predictions. However, time series of water consumption usually reveal a type of annual and weekly periodicity, which makes the time series not stationary and thus difficult to predict by regular prediction models. The annual periodicity cannot be directly extracted from the data, because it is usually irregular and related to the characteristic of a particular season in a particular year (early or late spring, dry or wet autumn, etc.), but it is strongly related to the weather conditions, so its influence on the prediction accuracy in more complex models can be reduced by adding the data describing current weather conditions. The weekly periodicity is more difficult to reduce, because it is also noised by the irregular annual periodicity.

**Figure 1:**
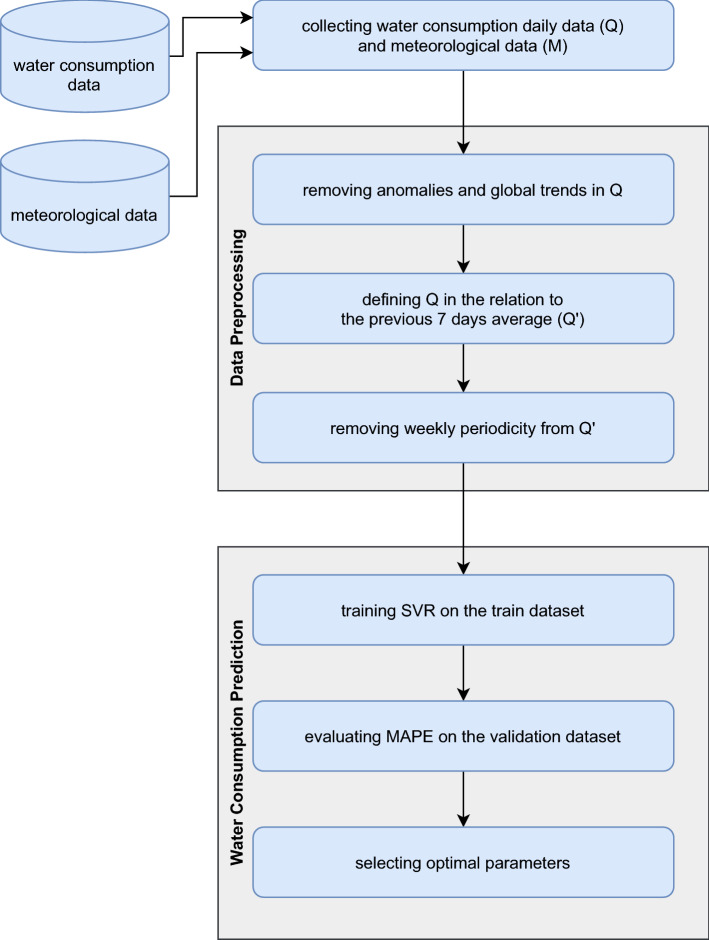
The methodology scheme.

In our approach, the input data for the model were water consumption and meteorological parameters (Fig. 1). First, the water consumption data (Q), obtained from measuring equipment and resampled to one-day frequency, were preprocessed by extracting anomalies and the global trend (defined by the 365-days moving average). Second, the water consumption data (Q) were expressed as a fraction (Q′) of the 7-days moving average (i.e., the average flow rate from the 7 days preceding each of the analyzed days), so that:

2 $$Q{^{\prime}}=\frac{Q}{{Q}_{7}}$$

where: $Q{^{\prime}}$—the current value of water consumption in relation to the previous 7 days average, $Q$—water consumption, ${\mathrm{Q}}_{7}$—the daily average of water consumption in the last 7 days.

Next, the relative water consumption data (Q′) were further preprocessed by extracting a weekly seasonality (evaluated either by the regular approach as a simple average of Mondays, Tuesdays, etc., or by the proposed evolutionary approach described further). Afterwards, the relative water consumption (Q′) was predicted using a linear regression model with the input data consisting of the values of Q′ from the previous 7 days and the weather data from the last day. Predictions were transformed to the original data scale by adding the weekly seasonality and the global trend and compared to the original data to evaluate the MAPE.

In the research, the prediction models were enhanced with evolutionary algorithms that discover the weekly periodicity in the optimization process. From the technical point of view, the weekly periodicity is a real number vector of length 7, and it is defined in a type of learning process as a solution to the optimization problem with the objective function being the accuracy of the prediction over a predefined training dataset and the search space consisting of real number vectors of length 7. To solve the optimization problem, an evolutionary algorithm based on an Evolution Strategy^56^ is proposed. It evolves a population of candidate solutions in the following process: first, the population includes a given number of random candidate solutions (random vectors from the search space drawn from the uniform probability distribution over the search space). Afterwards, the evolution starts and, in each iteration, a given number of parent candidate solutions is selected, then each pair of parent candidate solutions produces a pair of child candidate solutions, and finally, the selected child candidate solution replaces the previous candidate solution in the current population. Parent candidate solutions are selected with the probability proportional to their value of the objective function. Children are produced by local intermediary recombination and mutation operators, as in regular Evolution Strategies. The current population is updated with the best candidate solutions from the union of the current population and the child population. Finally, the evolution terminates after a given number of iterations.

**Figure 2:**
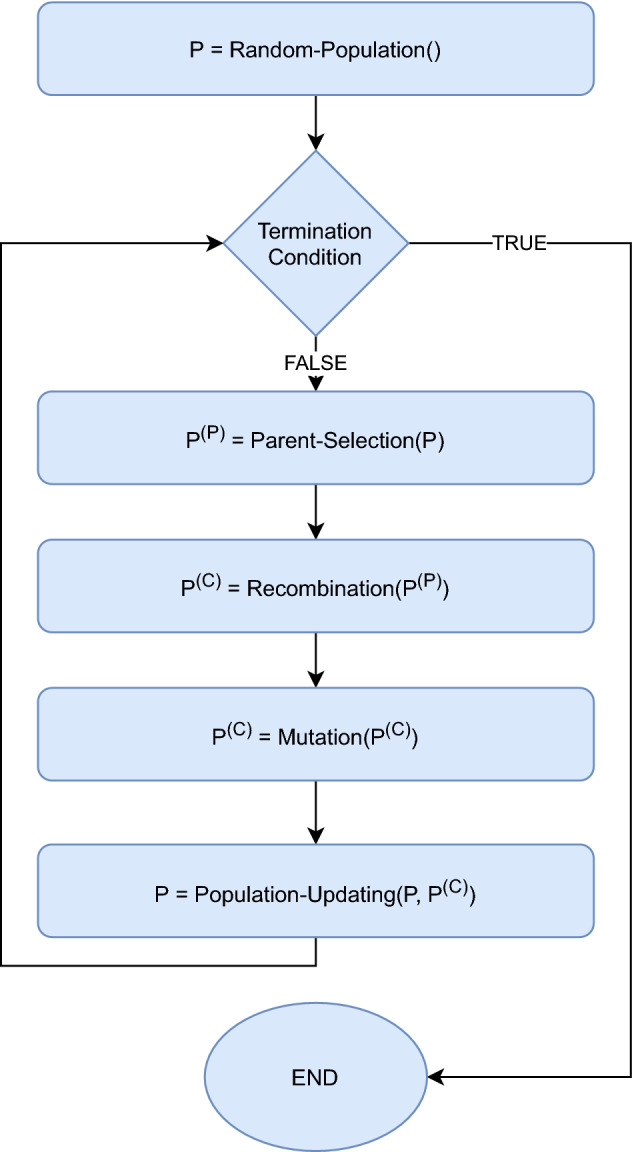
The general scheme of the evolutionary algorithm.

In the experiments, different configuration settings of the evolutionary algorithm were studied to calibrate the approach and the best results were obtained with the parent population size of 200, the offspring population size of 400, the number of iterations of 50 and other parameters set according to the general heuristics for Evolutionary Strategies^56^. Figure 2 presents the general schema of the algorithm.

### Consent for publication

All authors have given their permission to publish.

## Results and discussion

### Reference methods

In the first part of the research that was based on the standard approaches (SVR, MLP, LR, CART, and RF), it was assessed which of them allow to obtain the lowest possible value of the MAPE error, taking into account various variants of the input data. The learning process was carried out for all 10 datasets with information on historical water consumption (history0 and history1) and taking into account the optimal set of features. The implementation of all variables in the learning process could result in overfitting the model, therefore the aim of this part of the research is also to select the optimal set of input data. Since 12 features and extra features are blocked in 3 groups, there were 16,384 combinations and for every such combination the learning and evaluation procedure was performed.

Table 2 presents the results of the preliminary MAPE errors for the models created with the use of linear regression and for example, one dataset, taking into account various input data combinations.

**Table 2:** Mean absolute percentage error for linear regression and selected input data.

Feature subset description	MAPE (–)	Input data
Best combination without history	0.03662	Days of the week, wind speed, maximum temperature
All features	0.02814	All 15 features
Best one feature	0.02676	History0
Best two features	0.02463	History0, history1
Best for one week history	0.02405	History0, days of the week, precipitation, wind speed, minimum temperature
Best three features	0.02399	History0, history1, wind speed
Best without week days	0.02383	History0, history1, precipitation, wind speed, mean temperature
Best feature combination	0.02379	History0, history1, days of the week, precipitation, wind speed, ground temperature

Forecast errors ranging from 2.38 to 3.66% were obtained with the use of linear regression models. Out of all possible combinations of input data sets, the model with the best learning quality (MAPE equal to 2.38%) was obtained for the historical data, taking into account the water consumption level from the preceding 7 and 14 days, as well as a set of meteorological data: air humidity, wind speed and soil temperature. Due to the fact that some of the meteorological data are strongly correlated, e.g., soil temperature and air temperature, the use of all data does not improve the learning quality (MAPE error is 2.81%) and can lead to overfitting, even with such a simple approach as linear regression. The study results clearly show that the wind speed and historical values of water consumption are important in each case. Nevertheless, it should be emphasized that the most important factor is including the historical 7 and 14-day time series in the input data. The MAPE error for modelling with and without weather data differs by 0.08% (between the best two features datasets and the best without weekdays datasets) and 0.29% (between the best one feature and the best without weekday datasets). The meteorological parameters are strongly correlated with each other, therefore their selection for forecasting is biased.

Days of the week data were entered as the only input, apart from water consumption for modelling by, e.g., Bakker et al.^45^, where MAPE errors ranging from 1.44 to 5.12% were obtained for 24-h forecasts. Further research results, however, indicate that weather data significantly improve the model accuracy^17^. Antunes et al.^57^ based on their research indicate that the most important meteorological parameters when forecasting water consumption are: temperature, rain level, and rain occurrence, whereas Piasecki et al.^50^ claim, it is air humidity. Comparing the research results of other authors is not entirely reliable due to the fact that each of them has a different set of data, which depends on the local capabilities related to the possession of measuring devices as well as the forecasting scope. Pesantez et al.^51^ showed that for hourly forecasts, it is not necessary to have weather condition parameters due to their low variability within this time frame, but the research was carried out in relation to the users of the water supply network and not the entire DMA zone. The results of this research indicate that for daily or monthly forecasts, the information on the weather improves the accuracy of the model. The input data should include not only meteorological parameters, but most of all data informing about the historical variability of water consumption (7 and/or 14 days). They contain seasonality and variability caused mainly by human activity and its repetitive behavior in this area.

Due to the fact that the learning process was carried out on 10 fixed data sets, and one of the objectives was to investigate to what extent the selection of optimal features is random, a random forest regressor was used and the feature selection was performed multiple times. It was also checked if the feature selection is generally a good approach by comparing the performance of the regressors with all features and with a selected set. At the same time, it should be noted that the choice was made for one classifier and then tested on another. In the next stage of analyses, a water consumption model was created for the optimal set of features (history0, history1, days of the week, precipitation, wind speed, ground temperature) and the set of all features using methods more advanced than linear regression, i.e. with SVR, MPL and RF. This stage was aimed at indicating which of the methods will minimize MAPE and thus be implemented in the authors' evolutionary approach, with the optimal set of input data already selected. RF-k random forests with depth limited to K, Classification and Regression Trees (CART), MLPk-n Multi-Layer Perception regressor with k and n neurons in two hidden layers, and SVR-linear Support Vector regressor with linear kernel were used. Table 3 presents the obtained values of the MAPE forecast errors for the previously selected optimal variables and all features. Results are averaged over all 10 datasets, detailed results for each are presented in Appendix 1.

**Table 3:** Forecast mean absolute percentage error comparison for selected methods.

Methods	All features	Selected features
Linear Regression	0.02150	0.02197
SVR-linear	0.02481	0.02449
RF-5	0.02396	0.02408
RF-10	0.02404	0.02319
CART-Tree	0.03700	0.03356
MLP20-15	0.03986	0.03508
MLP30-10	0.04610	0.03162
MLP15-10	0.05010	0.02969
Average	0.03342	0.02796

The obtained study results indicate that the lowest MAPE error in the forecast of water consumption for all 10 datasets was obtained through Linear Regression and the largest for Multilayer Perceptron. This regularity applies both to the situation where all features were included in the input data and where those selected in the previous step were included. Random Forest method is the second that gives the lowest MAPE error after linear regression. The use of the most optimal set of features in the input model (history0, history1, days of the week, precipitation, wind speed, ground temperature) resulted in obtaining a smaller forecast error for practically most of the methods.

The obtained research results indicate that models based on linear regression are suitable for creating water demand forecasts without the need to implement more advanced solutions. After selecting the optimal set of features, even before implementing the evolutionary approach, which is the main goal of the research, the MAPE error is obtained at a satisfactory level of 2.2%.

### Evolutionary approach

This part of the article presents the results of research into the creation of a prognostic model containing implemented evolutionary algorithms, in which 10 selected datasets were used as testing data (similar to those in “Reference methods” section). The training data was a period of 1 year before each of the test datasets. To improve the quality of prediction, the evolutionary approach was implemented in conjunction with Linear Regression as the method through which the lowest MAPE error values were achieved.

Table 4 presents the prediction error for the prediction algorithm used in this experiment with the weekly periodicity vector determined by the evolutionary algorithm. MAPE values obtained during the implementation of the evolutionary approach for each of the ten datasets are presented. It includes both the quality of the forecasts for the training and testing sets.

**Table 4:** Prediction error for a method containing an evolutionary approach.

Test dataset			Dataset	
No.	Period		Training	Test
1	2016–01-28	2016–02-27	0.02054	0.01467
2	2016–04-22	2016–05-22	0.02000	0.02425
3	2016–09-15	2016–10-15	0.02055	**0.01314**
4	2016–11-05	2016–12-05	0.02051	0.01913
5	2017–10-31	2017–11-30	0.02008	0.01782
6	2018–06-01	2018–07-01	0.01889	0.01917
7	2018–07-21	2018–08-20	0.01806	**0.03306**
8	2019–01-16	2019–02-15	0.01981	0.01640
9	2019–06-01	2019–07-01	0.02019	0.02948
10	2019–10-27	2019–11-26	0.02093	0.02476
**MAPE (–)**			0.01996	0.02119

The best and the worst results on the test datasets are emphasised in bold.

The MAPE error for the training sets was 2.00%, while for the testing sets it was 2.12%. The results of the research show that the model with the implemented evolutionary approach allows to obtain water consumption forecasts with an error of only 1.31%. According to predictive model quality standards, a MAPE error below 10% should be regarded as a determinant of highly accurate forecasting^58^. Although it is not possible to directly compare the models created by other authors due to the different conditions and the scope of the conducted research, there are a number of results in which the MAPE values of forecasts range from 2.0 to 3.0%^17,37,50^ or they were containing larger error^49,57,59^. To make the obtained results more reliable, water consumption forecasts were intentionally made using standard methods (LR, SVR, MLP, RF, CART) on analogous data sets, which was described in the previous chapter. The implementation of evolutionary procedures allows to create short-term models of water consumption forecasts that are much more reliable, although the final value of the MAPE error is influenced by the measurement period and the range in which weekly periodicity was extracted from the time series.

**Figure 3:**
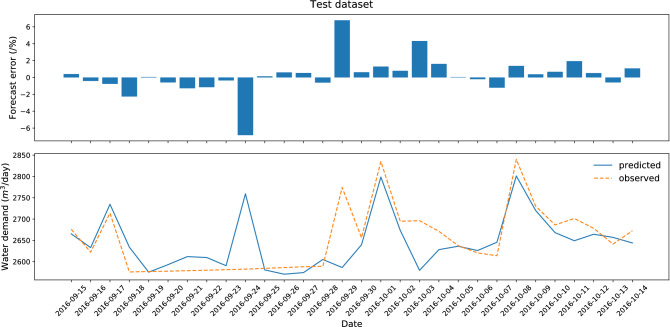
Research results for the best variant of forecasting—test dataset (dataset no. 3).

The results and diagrams for all ten datasets are included in the attachments, which constitute an appendix to the article (Appendix 2). The maximum likelihood model with a testing error of 1.31% was obtained for dataset no. 3 (Figs. 3. and 4). The testing process (30 successive days) ended with the greatest error in the case of September 24, 2016, when water consumption in the selected zone was almost 2.5 times higher than normal. It was caused by additional supply to the neighboring zone via the analyzed DMA. Similarly, on September 29, 2016, and October 3, 2016, the error value was significant due to a failure of the measuring device. All above-mentioned situations do not diminish the quality of the forecasting model due to the fact that they are random events.

**Figure 4:**
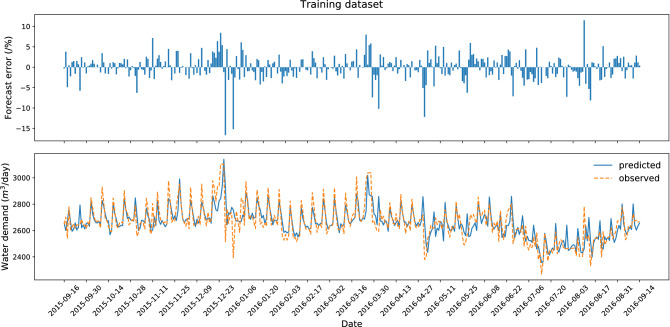
Research results for the best variant of forecasting—training dataset (dataset no. 3).

**Figure 5:**
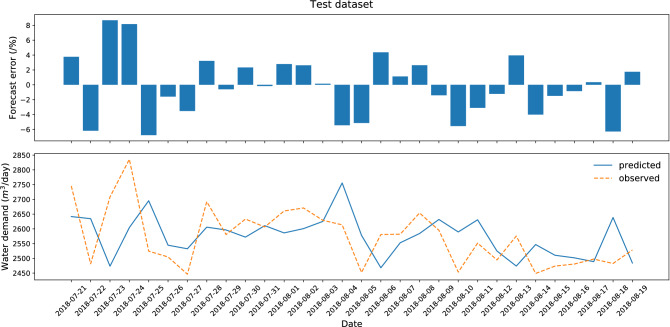
Research results for the worst variant of forecasting—test dataset (dataset no. 7).

**Figure 6:**
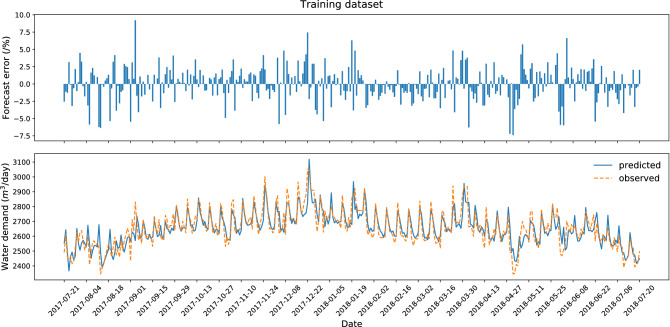
Research results for the worst variant of forecasting—training dataset (dataset no. 7).

The research results indicate that the worst accuracy of testing was obtained for dataset no. 7 from 2018-07-21 to 2018-08-20 (Figs. 5 and 6). The increase in the forecast error was caused by the record-breaking hot period, which resulted in increased consumption of tap water. This is another proof that weather conditions affect the forecasts of water consumption and its anomalies significantly affect the deterioration of the prediction.

**Figure 7:**
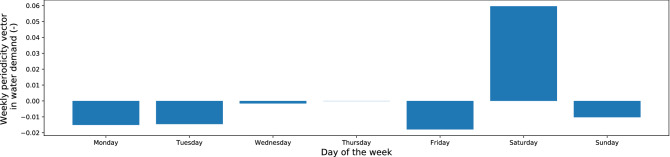
Weekly periodicity vector of water demand.

As defined in “Evolutionary approach” section, the weekly periodicity vector is discovered by an evolutionary algorithm as a solution to an optimization problem with the objective function that evaluates the accuracy of the candidate weekly periodicity vector on the training dataset. Figure 7 shows the distribution of weekly periodicity vector in water consumption. The evolutionary approach makes it possible to formulate some rules for water consumption by network users. It can be noticed that the greatest variability in water consumption is visible on Saturdays, while the lowest on Wednesdays. The results from end of the working week (Thursday–Friday) indicate that users of the water supply network are probably postponing household chores until Saturday. The water consumption habits of the inhabitants of DMA zones constitute a fairly large part of the research conducted by other scientists^22^ and the evolutionary approach may indirectly contribute to them and facilitate the creation of water demand patterns^28,60^.

**Figure 8:**
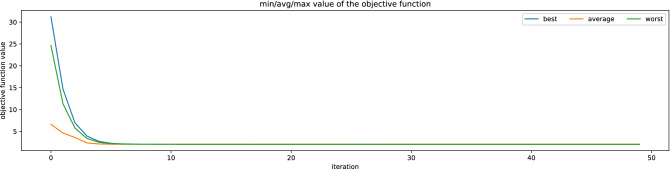
The evolution of the vectors of weekly periodicity in the successive iterations.

Figure 8 presents the objective function in the successive iterations of the evolutionary algorithm. It is easy to see that the evolutionary algorithm is capable of solving the optimization problem and discover a quasi-solution after about 5 iterations. A good learning algorithm should minimize the number of calculations required to achieve a good accuracy of the model^61^. For the learning process that models water demand, which is shaped by so many random factors, this is not a high number of iterations, which demonstrates that the proposed predicting methodology is effective.

**Figure 9:**
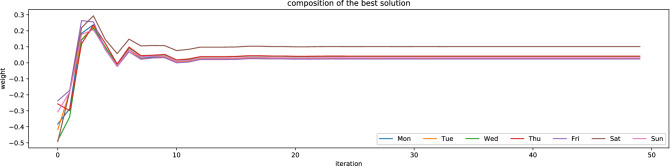
The evolution of the vectors of weekly periodicity in the successive iterations.

Figure 9 shows the evolution (changes in successive iterations of learning) of the weekly seasonality vector. In the chart, they are responsible for the consecutive days of the week. In the first iteration, the vector is random (not learned), while in the last iteration, it is the final vector. In intermediate iterations, it is noticeable how the evolution algorithm adjusts the vector values. For the first 10 or so iterations, the changes are quite large, then small, which means that the final vector was found quite quickly, later it was only slightly adjusted. There is an increased consumption of water on Saturdays, so the weight of the average water demand on a given weekday in relation to the weekly average is higher for Saturday.

## Conclusions and future work

Modern management of water supply infrastructure widely uses the development of information technologies that enable to enhance the processes connected with its operation. Time series of basic hydraulic parameters support increasingly advanced analytical and diagnostic tools. Water consumption prediction in various time horizons enables, among others, planning and performing maintenance and renovation works effectively, optimizing the pump station operations and detecting failures.

The research involved the implementation of a classical evolutionary approach for improving short-term prediction of water demand in one of the Wroclaw DMA zones with multifamily buildings. Additionally, an evolutionary strategy, which had not been used previously in this field of studies, was employed to determine the influence of weekly seasonality on water consumption distribution. The studies demonstrated the advantage of evolutionary strategies in combination with LR over standard methods of forecasting (SVR, MLP, RF, CART). The solution proposed by the authors enables to predict the volume of water consumption, expressed as the Mean Absolute Percentage Error (MAPE) more efficiently. The obtained final average values of MAPE errors were 2.12% (for the best 1.31% and worst 3.31% variant of forecasting, respectively). The research also demonstrated that evolutionary strategies are useful for data preprocessing and improving the knowledge of the anthropogenic behavior of water supply network users. Moreover, the results of the research indicate that the inclusion of additional variables in the input data in the form of meteorological parameters and water consumption for the preceding two weeks improves the accuracy of prediction.

In further studies, the authors intend to tackle the issues of predicting water demand in other DMA zones managed by the Municipal Water and Sewage Company, but of different consumption types, e.g., industrial consumption. An important element of the planned studies will be the spatial correlation of observations obtained from monitoring individual water supply areas.

## Data Availability

For data sources, see the Acknowledgments section; on analyses in this manuscript, please contact: justyna.stanczyk@upwr.edu.pl.

## Appendices

### Appendix 1: The results of the implementation of the references methods (MAPE)

**Table:**

Methods	Average	Set 1	Set 2	Set 3	Set 4	Set 5	Set 6	Set 7	Set 8	Set 9	Set 10
Linear regression	0.02197	0.01628	0.02329	0.01408	0.02201	0.01896	0.02024	0.03414	0.01675	0.02895	0.02500
SVR-linear	0.02449	0.01430	0.02184	0.02063	0.02806	0.02153	0.02240	0.03502	0.01996	0.02991	0.03126
RF-5	0.02408	0.02626	0.02204	0.01868	0.02340	0.02030	0.02493	0.03134	0.01701	0.03116	0.02570
RF-10	0.02319	0.02359	0.02211	0.01780	0.02551	0.01870	0.02254	0.03035	0.01663	0.02939	0.02533
CART-Tree	0.03356	0.03937	0.02674	0.03355	0.03578	0.02990	0.02995	0.03084	0.02347	0.05135	0.03466
MLP20-15	0.03508	0.04454	0.03069	0.02690	0.03521	0.03822	0.02439	0.03599	0.04117	0.03774	0.03592
MLP30-10	0.03162	0.02257	0.02337	0.03470	0.02322	0.02532	0.03753	0.03745	0.02285	0.04982	0.03933
MLP15-10	0.02969	0.02444	0.02445	0.02669	0.02540	0.02656	0.03170	0.04073	0.03646	0.02603	0.03443

### Appendix 2: The results of the implementation of the evolutionary approach

#### Test datasets

Dataset no. 1

Dataset no. 2

Dataset no. 3

Dataset no. 4

Dataset no. 5

Dataset no. 6

Dataset no. 7

Dataset no. 8

Dataset no. 9

Dataset no. 10.

#### Training datasets

Dataset no. 1

Dataset no. 2

Dataset no. 3

Dataset no. 4

Dataset no. 5

Dataset no. 6

Dataset no. 7

Dataset no. 8

Dataset no. 9

Dataset no. 10

## References

[1] Haddeland I (2014). Global water resources affected by human interventions and climate change. PNAS.

[2] Hussain Z (2022). A comparative appraisal of classical and holistic water scarcity indicators. Water Resour. Manag..

[3] Rinaudo, J.-D. Long-term water demand forecasting. Understanding and managing urban water in transition. 239–268 (2015).

[4] Shirkoohi MG, Doghri M, Duchesne S (2021). Short-term water demand predictions coupling an artificial neural network model and a genetic algorithm. Water Supply.

[5] Candelieri A, Giordani I, Archetti F, Barkalov K, Meyerov I, Polovinkin A, Sysoyev A, Zolotykh N (2019). Tuning hyperparameters of a SVM-based water demand forecasting system through parallel global optimization. Comput. Oper. Res..

[6] Brentan BM, Luvizotto E, Herrera M, Izquierdo J, Pérez-García R (2017). Hybrid regression model for near real-time urban water demand forecasting. J. Comput. Appl. Math..

[7] Tiwari MK, Adamowski JF (2015). Medium-term urban water demand forecasting with limited data using an ensemble wavelet–bootstrap machine-learning approach. J. Water Resour. Plan. Manag..

[8] Candelieri A, Soldi D, Archetti F (2015). Layered machine learning for short-term water demand forecasting. Eng. Manag. J..

[9] Candelieri A, Soldi D, Archetti F (2015). Short-term forecasting of hourly water consumption by using automatic metering readers data. Procedia Eng..

[10] Ghiassi M, Fa’al F, Abrishamchi A (2017). Large metropolitan water demand forecasting using DAN2, FTDNN, and KNN models: A case study of the city of Tehran, Iran. Urban Water J..

[11] Hemati A, Rippy MA, Grant SB, Davis K, Feldman D (2016). Deconstructing demand: The anthropogenic and climatic drivers of urban water consumption. Environ. Sci. Technol..

[12] Guyennon N, Romano E, Portoghese I (2016). Long-term climate sensitivity of an integrated water supply system: The role of irrigation. Sci. Total Environ..

[13] Berglund EZ, Monroe JG, Ahmed I, Noghabaei M, Pesantez JE, Khaksar Fasaee MA, Bardaka E, Han K, Proestos G, Levis J (2020). State-of-the-art review: smart infrastructure: A vision for the role of the civil engineering profession in smart cities. J. Infrastruct. Syst..

[14] Dawood T, Elwakil E, Novoa HM, Delgado JFG (2021). Ensemble intelligent systems for predicting water network condition index. Sustain. Cities Soc..

[15] Novak J, Melenhorst M, Micheel I, Pasini C, Fraternali P, Rizzoli AE (2018). Integrating behavioural change and gamified incentive modelling for stimulating water saving. Environ. Model. Softw..

[16] Tang K, Parsons DJ, Jude S (2019). Comparison of automatic and guided learning for Bayesian networks to analyse pipe failures in the water distribution system. Reliab. Eng. Syst. Saf..

[17] Bakker M, Van Duist H, Van Schagen K, Vreeburg J, Rietveld L (2014). Improving the performance of water demand forecasting models by using weather input. Procedia Eng..

[18] Sebri M (2016). Forecasting urban water demand: A meta-regression analysis. J. Environ. Manag..

[19] Eliades DG, Polycarpou MM (2012). Leakage fault detection in district metered areas of water distribution systems. J. Hydroinformatics.

[20] Ambrosio JK, Brentan BM, Herrera M, Luvizotto E, Ribeiro L, Izquierdo J (2019). Committee machines for hourly water demand forecasting in water supply systems. Math. Probl. Eng..

[21] Pacchin E, Gagliardi F, Alvisi S, Franchini M (2019). A comparison of short-term water demand forecasting models. Water Resour. Manag..

[22] Vieira P, Jorge C, Covas D (2017). Assessment of household water use efficiency using performance indices. Resour. Conserv. Recycl..

[23] Zangenehmadar Z, Moselhi O (2016). Prioritizing deterioration factors of water pipelines using Delphi method. Measurement.

[24] Arsene CT, Gabrys B (2014). Mixed simulation-state estimation of water distribution systems based on a least squares loop flows state estimator. Appl. Math. Model..

[25] Tavakoli A, Rahimpour M (2014). Gröbner bases for solving ΔQ-equations in water distribution networks. Appl. Math. Model..

[26] Zubaidi SL (2020). A novel methodology for prediction urban water demand by wavelet denoising and adaptive neuro-fuzzy inference system approach. Water.

[27] Alvisi S, Franchini M (2014). Assessment of the predictive uncertainty within the framework of water demand forecasting by using the model conditional processor. Procedia Eng..

[28] Candelieri A, Archetti F (2014). Identifying typical urban water demand patterns for a reliable short-term forecasting–the icewater project approach. Procedia Eng..

[29] Chen J, Boccelli D (2014). Demand forecasting for water distribution systems. Procedia Eng..

[30] Kofinas D, Mellios N, Papageorgiou E, Laspidou C (2014). Urban water demand forecasting for the island of Skiathos. Procedia Eng..

[31] Romano M, Kapelan Z (2014). Adaptive water demand forecasting for near real-time management of smart water distribution systems. Environ. Model. Soft..

[32] Tiwari M, Adamowski J, Adamowski K (2016). Water demand forecasting using extreme learning machines. J. Water Land Dev..

[33] Ernesto A, Amadou Ba, Bradley E, Sean McKenna (2016). Tailoring seasonal time series models to forecast short-term water demand. J. Water Resour. Plan. Manag..

[34] Duerr I, Merrill HR, Wang C, Bai R, Boyer M, Dukes MD, Bliznyuk N (2018). Forecasting urban household water demand with statistical and machine learning methods using large space-time data: A comparative study. Environ. Model. Softw..

[35] Kozłowski E, Kowalska B, Kowalski D, Mazurkiewicz D (2018). Water demand forecasting by trend and harmonic analysis. Arch. Civ. Mech. Eng..

[36] Xenochristou M, Kapelan Z, Hutton C, Hofman J (2018). Smart water demand forecasting: Learning from the data. EPiC Ser. Eng..

[37] Xu Y, Zhang J, Long Z, Tang H, Zhang X (2019). Hourly urban water demand forecasting using the continuous deep belief echo state network. Water.

[38] Guo W, Liu T, Dai F, Xu P (2020). An improved whale optimization algorithm for forecasting water resources demand. Appl. Soft Comput..

[39] Karamaziotis PI, Raptis A, Nikolopoulos K, Litsiou K, Assimakopoulos V (2020). An empirical investigation of water consumption forecasting methods. Int. J. Forecast..

[40] Smolak K, Kasieczka B, Fialkiewicz W, Rohm W, Siła-Nowicka K, Kopańczyk K (2020). Applying human mobility and water consumption data for short-term water demand forecasting using classical and machine learning models. Urban Water J..

[41] Bata M, Carriveau R, Ting DS-K (2020). Short-term water demand forecasting using hybrid supervised and unsupervised machine learning model. Smart Water.

[42] Bata M, Carriveau R, Ting DS-K (2020). Short-term water demand forecasting using nonlinear autoregressive artificial neural networks. J. Water Resour. Plan. Manag..

[43] Okeya I, Kapelan Z, Hutton C, Naga D (2014). Online burst detection in a water distribution system using the Kalman filter and hydraulic modelling. Procedia Eng..

[44] Wu Y, Liu S, Wu X, Liu Y, Guan Y (2016). Burst detection in district metering areas using a data driven clustering algorithm. Water Res..

[45] Bakker M, Vreeburg J, Van Schagen K, Rietveld L (2013). A fully adaptive forecasting model for short-term drinking water demand. Environ. Model. Softw..

[46] Zhou SL, McMahon TA, Walton A, Lewis J (2002). Forecasting operational demand for an urban water supply zone. J. Hydrol..

[47] Statistical Office in Wrocław, 2017. Wrocław in Figures. http://wroclaw.stat.gov.pl/publikacje-i-foldery/foldery/wroclaw-w-liczbach-2017-folder,1,4.html (Accessed 29 Sept 2017).

[48] Maruyama Y, Yamamoto H (2019). A study of statistical forecasting method concerning water demand. Procedia Manuf..

[49] Velasco, L., Granados, A., Ortega, J. & Pagtalunan, K. Medium-term water consumption forecasting using artificial neural networks. Presented at the 17th Conference of the Science Council of Asia, National Research Council of the Philippines (2017)

[50] Piasecki A, Jurasz J, Kaźmierczak B (2018). Forecasting daily water consumption: A case study in Torun, Poland. Period. Polytech.-Civ..

[51] Pesantez JE, Berglund EZ, Kaza N (2020). Smart meters data for modeling and forecasting water demand at the user-level. Environ. Model. Softw..

[52] Murphy KP (2012). Machine learning: a probabilistic perspective.

[53] Goodfellow, I., Bengio, Y. & Courville, A. *Deep Learning (Adaptive Computation and Machine Learning Series)*. 321–359 (2017)

[54] Scikit-learn, machine learning in Python. https://scikit-learn.org.

[55] Moreno JJM, Pol AP, Abad AS, Blasco BC (2013). Using the R-MAPE index as a resistant measure of forecast accuracy. Psicothema.

[56] Kramer O (2016). Machine Learning for Evolution Strategies.

[57] Antunes A, Andrade-Campos A, Sardinha-Lourenço A, Oliveira M (2018). Short-term water demand forecasting using machine learning techniques. J. Hydroinformatics..

[58] Lewis CD (1982). Industrial and Business Forecasting Methods: A Practical Guide to Exponential Smoothing and Curve Fitting.

[59] Benítez R, Ortiz-Caraballo C, Preciado JC, Conejero JM, Sánchez Figueroa F, Rubio-Largo A (2019). A short-term data based water consumption prediction approach. Energies.

[60] Alvisi S, Franchini M, Marinelli A (2007). A short-term, pattern-based model for water-demand forecasting. J. Hydroinformatics..

[61] Joachims, T. Making large-scale SVM learning practical. Technical report (1998) https://www.econstor.eu/handle/10419/77178.

